# Predictors of screen viewing time in young Singaporean children: the GUSTO cohort

**DOI:** 10.1186/s12966-017-0562-3

**Published:** 2017-09-05

**Authors:** Jonathan Y. Bernard, Natarajan Padmapriya, Bozhi Chen, Shirong Cai, Kok Hian Tan, Fabian Yap, Lynette Shek, Yap-Seng Chong, Peter D. Gluckman, Keith M. Godfrey, Michael S. Kramer, Seang Mei Saw, Falk Müller-Riemenschneider

**Affiliations:** 1 Singapore Institute for Clinical Sciences, Agency for Science, Technology and Research (A*STAR), Singapore, Singapore; 2 Department of Obstetrics and Gynaecology, Yong Loo Lin School of Medicine, National University of Singapore, Singapore, Singapore; 3 Saw Swee Hock School of Public Health, National University of Singapore, Singapore, Singapore; 4 Department of Maternal Fetal Medicine, KK Women’s and Children’s Hospital, Singapore, Singapore; 5 Duke-NUS Medical School, Singapore, Singapore; 6 Department of Paediatrics, KK Women’s and Children’s Hospital, Singapore, Singapore; 7 Department of Paediatrics, Yong Loo Lin School of Medicine, National University of Singapore, Singapore, Singapore; 8 Division of Paediatric Allergy, Immunology and Rheumatology, Khoo Teck Puat - National University Children’s Medical Institute, National University Health System, Singapore, Singapore; 9 Liggins Institute, University of Auckland, Auckland, New Zealand; 10 Medical Research Council Lifecourse Epidemiology Unit, University of Southampton, Southampton, UK; 11 NIHR Southampton Biomedical Research Centre, University of Southampton and University Hospital Southampton NHS Foundation Trust, Southampton, UK; 12 Departments of Pediatrics and of Epidemiology and Biostatistics, McGill University Faculty of Medicine, Montreal, Quebec, Canada; 13 Singapore Eye Research Institute, Singapore, Singapore; 14 Institute for Social Medicine, Epidemiology and Health Economics, Charite University Medical Centre, Berlin, Germany; 15 Singapore Institute for Clinical Sciences (SICS), Agency for Sciences, Technology and Research (A*STAR). MD1 Tahir Foundation Building, #12-02/03, 21 Lower Kent Ridge Rd, Singapore, 119077 Singapore

**Keywords:** Television, Computer, Hand-held device, Sedentary lifestyle, Childhood, Cohort studies

## Abstract

**Background:**

Higher screen viewing time (SVT) in childhood has been associated with adverse health outcomes, but the predictors of SVT in early childhood are poorly understood. We examined the sociodemographic and behavioral predictors of total and device-specific SVT in a Singaporean cohort.

**Methods:**

At ages 2 and 3 years, SVT of 910 children was reported by their parents. Interviewer-administered questionnaires assessed SVT on weekdays and weekends for television, computer, and hand-held devices. Multivariable linear mixed-effect models were used to examine the associations of total and device-specific SVT at ages 2 and 3 with predictors, including children’s sex, ethnicity, birth order, family income, and parental age, education, BMI, and television viewing time.

**Results:**

At age 2, children’s total SVT averaged 2.4 ± 2.2 (mean ± SD) hours/day, including 1.6 ± 1.6 and 0.7 ± 1.0 h/day for television and hand-held devices, respectively. At age 3, hand-held device SVT was 0.3 (95% CI: 0.2, 0.4) hours/day higher, while no increases were observed for other devices. SVT tracked moderately from 2 to 3 years (*r* = 0.49, *p* < 0.0001). Compared to Chinese children, Malay and Indian children spent 1.04 (0.66, 1.41) and 0.54 (0.15, 0.94) more hours/day watching screens, respectively. Other predictors of longer SVT were younger maternal age, lower maternal education, and longer parental television time.

**Conclusions:**

In our cohort, the main predictors of longer children’s SVT were Malay and Indian ethnicity, younger maternal age, lower education and longer parental television viewing time. Our study may help target populations for future interventions in Asia, but also in other technology-centered societies.

**Trial registration:**

This ongoing study was first registered on July 1, 2010 on NCT01174875 as. Retrospectively registered.

**Electronic supplementary material:**

The online version of this article (doi:10.1186/s12966-017-0562-3) contains supplementary material, which is available to authorized users.

## Background

In the 1960’s, television became widely introduced into homes of high-income countries and rapidly occupied a substantial fraction of individuals’ leisure time. In 2013, watching television reached an average of 3.5 h daily in US adults, i.e., about 10 years total accumulated over the life course [1]. Since the 1980’s, office-based workers have spent increasing part of their work day facing computer monitors, and more recently, playing video games has become a popular home activity for many Western adults and children [2–4]. Screens have now evolved to pocket, mobile, and personal devices, such as tablets and smartphones, which broaden the opportunities to use such devices in different locations and times. Importantly, hand-held devices have become increasingly accessible to young children and to individuals from lower socioeconomic strata and those from low- and middle-income countries.

Lack of physical activity is now recognized as a leading cause of preventable morbidity and mortality globally [5–7]. Less evidence is available, however, regarding high levels of sedentary behavior, which differs from inactivity [5, 8]. In adults, a sedentary lifestyle is an independent risk factor for cardiovascular and metabolic diseases [9]. In childhood, sedentary lifestyle has been independently associated with obesity, higher blood pressure and poorer mental health [9]. This level of evidence remains fragile, however, owing to cross-sectional designs, the complexity of accurately measuring sedentary behavior in young children, and the potential for residual confounding. Screen use is a waking activity involving low energy expenditure and is therefore considered a form of sedentary behavior [8, 10]. Screen viewing time (SVT) in preschool children is associated with eating disorders, reduced sleep duration, development delays, attention deficit and myopia [11–13]. As early as age 2, SVT has been associated with childhood obesity [14]. Based on this evidence, the American Academy of Pediatrics (AAP) now recommends that children below 18 months avoid any digital media use but suggests the gradual introduction of family-shared, high-quality content between 18 months and 2 years, while limiting screen time to a maximum of 1 h/day between 2 and 5 years of age [15].

Populations living in high-income Asian countries have become among the top users of all kinds of screen devices. According to the Google Consumer Barometer survey, in 2015 only 6% of persons ≥16 years living in South Korea or Singapore owned no devices (vs 13% in the U.S.) [16]. This survey also reported that 91% of Singaporeans owned a smartphone, 41% a tablet, and 60% at least 3 screen devices. The above statistics on prevalence of SVT have remained fairly stable in Singapore over recent years but are rising rapidly in Western countries [16]. In some ways, observations from “high-tech” Asian countries may presage a similar situation in Western populations. A multiscreen environment may encourage screen use in early childhood. To date, predictors of screen viewing behavior in Asian children are not well known; they may differ from those reported in other populations [17, 18]. As an illustration, a recent Singaporean cross-sectional study focusing on device-specific screen use in children less than 2 years of age reported a median number of 2 televisions, 2 computers, and 4 hand-held devices in their homes, while half of their parents had a total SVT ≥8 h daily [19]. Parents of less than half the sample of children aged <2 years reported no screen viewing, while 16% reported ≥2 h daily. Ethnicity, parental screen viewing and rule setting practices were identified as independent predictors of total SVT [19]. Longitudinal data are needed to establish the temporal precedence of potential predictors of SVT and changes in SVT over time. We thus aimed to describe total and device-specific SVT and assess its predictors, both cross-sectionally and longitudinally, among children aged 2 and 3 years enrolled in a tri-ethnic Singaporean cohort.

## Methods

### Study design and population

Between June 2009 and September 2010, the Growing Up in Singapore Towards healthy Outcomes (GUSTO) study recruited pregnant women of Chinese, Malay and Indian ethnicities who visited two public maternity units (KK Hospital and National University Hospital) in Singapore for their first ultrasound scan before the 15th week of pregnancy. The main exclusion criteria were non-homogeneous ethnic background (up to the four grandparents of the offspring), intention not to deliver in the study centers or not to remain in Singapore for the following 5 years. From the 1247 pregnant women recruited, 1171 singleton newborns were included and followed regularly. The recruitment and follow-up protocol has been detailed previously [20]. All participants signed written informed consent at enrolment. The study received ethical approval from the National Healthcare Group Domain Specific Review Board and the SingHealth Centralised Institutional Review Board.

### Data collection

Sociodemographic and health information was obtained at enrolment as part of an interviewer-administered questionnaire: ethnicity (Chinese/Malay/Indian), maternal and paternal age, maternal highest education level (secondary or less/post-secondary/university), marital status (single/married), maternal place of birth (Singapore/other), accommodation type (public/private housing), monthly household income (<2000/2000-3999/4000-5999/≥6000 Singapore dollars), and maternal pre-pregnancy weight (kg). During the clinic visit at 26-28 weeks’ gestation, maternal height (cm) was measured, and pre-pregnancy and pregnancy behavioral information was obtained through an interviewer-administered questionnaire: tobacco (yes/no) and alcohol consumption (yes/no), television viewing time (6 categories from <1 h to >5 h daily) and physical activity (frequency and duration of light-moderate, moderate and vigorous intensity activities). Information on the offspring’s date of birth, sex and birth order was extracted from medical records. Paternal data were obtained from the fathers when they attended (80% of them did) postnatal visits with their child at 24 or 36 months: highest education level and television viewing time (same categories as for mothers) via interviewer-administered questionnaires, while weight (kg) and height (cm) were both measured. Time point and measurement method of the collected data are summarized in Additional file 1: Table S1.

Maternal and paternal age were categorized into 4 groups: <25, 25-29, 30-34 and ≥35 years for mothers and <30, 30-34, 35-39, and ≥40 years for fathers. Maternal pre-pregnancy and paternal BMI was derived from weight divided by height squared (m^2^), then categorized according to WHO criteria (<18.5, 18.5-24.9, 25.0-29.9, and ≥30.0 kg/m^2^). Maternal and paternal television viewing times were categorized as <1 h, 1-2 h, 2-3 h or ≥3 h/day. Maternal physical activity before pregnancy was categorized as insufficiently, sufficiently or highly active, as detailed previously [21].

### Screen viewing time (SVT)

Screen use data were assessed at the 2- and 3-year clinic visits as part of a questionnaire on quantitating children’s time spent in indoor and outdoor activities. Trained interviewers asked the parents how much time, in 5-min increments, their child spent on average using screens on weekdays and weekend days. Three types of devices (with examples enunciated to the participants) were considered: 1) television and television games (e.g., PlayStation®, Wii™, Xbox™), further referred to as ‘television,’ 2) computers, and 3) hand-held video games and hand phones (e.g., Game Boy®, hand-phone games), including tablets, hereinafter referred to as hand-held devices. Although the item ‘computer’ was enunciated without example, the interviewers were trained to clarify, if necessary, that it includes both desktop and laptop computers. For each type, weekday and weekend day times were averaged to obtain device-specific SVT in hours/day ([weekday × 5 + weekend day × 2]/7). Total SVT was calculated as the sum of the times for the three types of device.

### Statistical analyses

Total and device-specific SVT at 2 and 3 years, and the changes between those ages, are described as mean ± standard deviation (SD) and median and interquartile range (IQR), while categorical variables are summarized using frequencies and percentages. Spearman’s correlation was used to assess the degree of SVT tracking between 2 and 3 years.

**Fig. 1:**
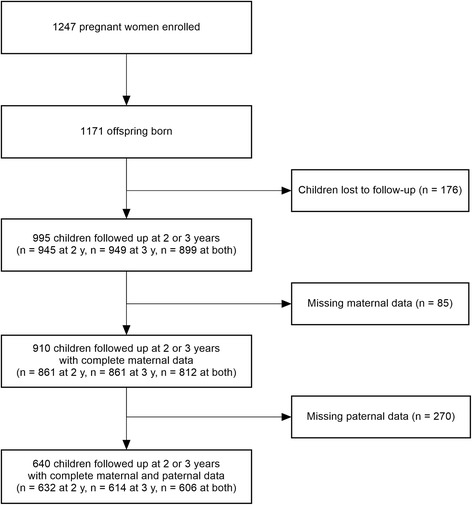
Flow diagram of the GUSTO study participants followed up to 2 and 3 years

Potential predictors of SVT at 2 and 3 years were examined in several steps. First, parental and child predictors were screened by linear regression to identify those associated with SVT in unadjusted models. Second, multivariable linear regression models were built using a forward selection approach and *p* ≤ 0.25 as an entering criterion. To maximize statistical power, multivariable analyses were restricted to the sample with complete data for both the offspring and the mother (*n* = 910), but not necessarily with complete paternal data (since they were missing for *n* = 270). Multivariable models with the paternal variables were built afterwards on the sample with complete data for the three family members (*n* = 640). We also carried out a sensitivity analysis using multiple imputation for missing paternal data. Third, repeated measures linear regression model with an unstructured variance-covariance matrix was used to account for non-independence among the repeated outcomes for each individual assessed at both time points. This also permitted us to use a single model for children whose SVT was estimated at one time point only (see Fig. 1). Interactions between the predictors and time points were tested to evaluate whether predictors were associated differentially with SVT at 2 and 3 years. Significant interaction denotes an association between the predictor and the change in SVT from 2 to 3 years [22], but has the additional advantage of including all children assessed at either age; *p*-interaction was considered significant when <0.10, since tests of interaction tend to be statistically under-powered. Interactions between child sex and ethnicity, and child sex and parental television time were also examined. Finally, we used multivariable logistic regression to examine whether the predictors of total SVT also predicted the odds of ≥2 and ≥4 h/day. All statistical analyses were carried out with SAS 9.4 (SAS Institute Inc., Cary, NC, USA) during the second half of 2016.

## Results

Overall, 910 parents reported their child’s SVT, including 812 reporting SVT at both ages (*n* = 812) and those who reported at 2 years only (*n* = 49) or 3 years only (*n* = 49) (Fig. 1). At age 2 years, the children spent an average of 2.4 ± 2.2 h of total SVT, mainly contributed by 1.6 ± 1.7 h of television and 0.7 ± 1.0 of hand-held devices (Table 1). Fewer children engaged in any computer use: 21 and 18% at 2 and 3 years, respectively. Thus, computer use was not examined further separately. At age 3 years, total SVT increased on average by 0.33 ± 2.42 h/day, mainly owing to an increase in hand-held device viewing time. SVTs at 2 and 3 years were moderately correlated (*r* = 0.45 for television, *r* = 0.42 hand-held devices, *r* = 0.49 for total SVT, all *p* < 0.0001, not shown).

**Table 1:** Daily total and device-specific SVT in 2- and 3-year-old children from the GUSTO cohort study

	*n* (%)	Mean ± SD	Median (IQR)
Screen viewing time at 2 years	861^a^		
Television and video game, *hour/day*		1.6 ± 1.7	1.0 (0.5 – 2.3)
Computer, *hour/day*		0.1 ± 0.4	0.0 (0.0 – 0.0)
Hand-held devices, *hour/day*		0.7 ± 1.0	0.3 (0.1 – 0.9)
Total, hour/day		2.4 ± 2.2	1.9 (0.9 – 3.3)
<2 h/day	439 (51.0)		
2-4 h/day	257 (29.9)		
≥4 h/day	165 (19.2)		
Screen viewing time at 3 years	861^a^		
Television and video game, *hour/day*		1.6 ± 1.5	1.0 (0.5 – 2.3)
Computer, *hour/day*		0.1 ± 0.4	0.0 (0.0 – 0.0)
Hand-held devices, *hour/day*		0.9 ± 1.2	0.5 (0.2 – 1.0)
Total, *hour/day*		2.7 ± 2.2	2.1 (1.1 – 3.6)
<2 h/day	387 (45.0)		
2-4 h/day	274 (31.8)		
≥4 h/day	200 (23.2)		
Change between 2 and 3 years	812		
Television and video game, *hour/day*		0.03 ± 1.82	0.0 (−1.0 – 1.0)
Computer, *hour/day*		0.01 ± 0.56	0.0 (0.0 – 0.0)
Hand-held devices, *hour/day*		0.29 ± 1.45	0.0 (0.0 – 1.0)
Total, *hour/day*		0.33 ± 2.42	0.0 (−1.0 – 1.0)

^a^Maximum sample size with data at each age (sample overlapping both ages = 812)

The study sample at 2 and 3 years of age is described in Table 2. Predictors of child SVT that passed the multivariable model selection criteria were ethnicity, household income, parental age and education, and higher maternal television viewing time (all *p* < 0.001). Marital status, maternal place of birth, accommodation type, maternal tobacco and alcohol consumption, and parental BMI did not reach the model selection criteria (*p* > 0.25, not shown) and were therefore excluded from the subsequent multivariable models. Although not associated with SVT, child sex and exact age were forced into the models.

**Table 2:** Sample description in 2- and 3-year-old children

	Subsample assessed at 2 years (*n* = 861)	Subsample assessed at 3 years (*n* = 861)
Child age, months	24.4 ± 0.9	36.5 ± 1.1
Study centre		
KKH	654 (76.0)	651 (75.6)
NUH	207 (24.0)	210 (24.4)
Ethnicity		
Chinese	490 (56.9)	492 (57.1)
Malay	220 (25.6)	219 (25.4)
Indian	151 (17.5)	150 (17.4)
Child sex		
Male	457 (53.1)	461 (43.5)
Female	404 (46.9)	400 (46.5)
Birth order		
First-born	383 (44.5)	387 (45.0)
Second- or later-born	478 (55.5)	474 (55.1)
Monthly household incomes		
< 2000 SGD	123 (14.3)	126 (14.6)
2000-3999 SGD	262 (30.4)	253 (29.4)
4000-5999 SGD	218 (25.3)	212 (24.6)
≥ 6000 SGD	258 (30.0)	270 (31.4)
Accommodation type		
Public housing	786 (91.3)	787 (91.4)
Private	75 (8.7)	74 (8.6)
Maternal place of birth		
Singapore	539 (62.6)	542 (63.0)
Abroad	322 (37.4)	319 (37.1)
Maternal age		
< 25 years	83 (9.6)	82 (9.5)
25-29 years	263 (30.6)	259 (30.1)
30-34 years	279 (32.4)	283 (32.9)
≥ 35 years	236 (27.4)	237 (27.5)
Maternal education		
University	300 (34.8)	309 (35.9)
Post-secondary	302 (35.1)	294 (34.2)
Primary or Secondary	259 (30.1)	258 (30.0)
Maternal BMI before pregnancy, kg/m^2^	22.8 ± 4.4	22.8 ± 4.4
Maternal tobacco consumption before pregnancy		
Yes	92 (10.7)	89 (10.3)
No	769 (89.3)	772 (89.7)
Maternal alcohol consumption before pregnancy		
Yes	295 (34.3)	297 (34.5)
No	566 (65.7)	564 (65.5)
Maternal physical activity level before pregnancy		
Insufficiently active	281 (32.9)	286 (33.5)
Sufficiently active	419 (49.1)	418 (48.9)
Highly active	153 (17.9)	151 (17.7)
Missing, *n*	8	6
Maternal daily television-viewing time		
< 1 h	152 (17.7)	154 (17.9)
1-2 h	237 (27.5)	235 (27.3)
2-3 h	187 (21.7)	195 (22.7)
≥ 3 h	285 (33.1)	277 (32.2)
Maternal marital status		
Married	829 (97.1)	828 (97.1)
Single/divorced	25 (2.9)	25 (2.9)
Missing, *n*	7	8
Paternal age		
< 30 years	164 (22.0)	156 (21.5)
30-34 years	243 (32.6)	235 (32.3)
35-39 years	209 (28.0)	207 (28.5)
≥ 40 years	130 (17.4)	129 (17.7)
Missing, *n*	115	134
Paternal education		
University	279 (39.0)	277 (39.6)
Post-secondary	157 (22.0)	151 (21.6)
Primary or Secondary	279 (39.0)	272 (38.9)
Missing, *n*	146	161
Paternal BMI^a^, kg/m^2^	25.7 ± 4.5	25.9 ± 4.8
Paternal daily television-viewing time		
< 1 h	235 (32.9)	236 (33.7)
1-2 h	268 (37.5)	256 (36.6)
2-3 h	102 (14.3)	103 (14.7)
≥ 3 h	110 (15.4)	105 (15.0)
Missing, *n*	146	161

Values are Mean ± SD or % (n) for continuous and categorical variables, respectively

^a^Missing data: 63 and 61 at in 2-year and 3-year samples, respectively

Results of linear mixed-effect regression models adjusted for maternal data (model 1) and both parents’ data (model 2) are shown in Table 3. Compared to Chinese children, Malay and Indian children spent 0.94 (0.64, 1.25) and 0.60 (0.28, 0.92) more hours/day, respectively, in total SVT. Other sociodemographic variables independently associated with total or device-specific SVT were birth order, low household income, maternal education less than university level and paternal age < 35 years. Longer maternal and paternal television viewing times were associated with longer total SVT.

**Table 3:** Adjusted associations between predictors and total and device-specific SVT (h/day) in 2- and 3-year-old children

	Total screen viewing time		TV viewing time		Hand-held device viewing time	
	Model 1	Model 2	Model 1	Model 2	Model 1	Model 2
	B (95% CI)	B (95% CI)	B (95% CI)	B (95% CI)	B (95% CI)	B (95% CI)
Intercept	1.22 (0.80, 1.64)	0.95 (0.39, 1.51)	0.75 (0.44, 1.05)	0.61 (0.19, 1.03)	0.37 (0.16, 0.58)	0.24 (−0.04, 0.51)
Age (ref: 2-year)						
3-year	0.29 (0.13, 0.46)	0.29 (0.10, 0.48)	0.03 (−0.09, 0.15)	0.00 (−0.13, 0.14)	0.27 (0.18, 0.36)	0.30 (0.20, 0.41)
Centre (ref: KKH)						
NUH	0.22 (−0.05, 0.50)	0.06 (−0.28, 0.39)	0.27 (0.07, 0.47)	0.18 (−0.07, 0.43)	−0.06 (−0.20, 0.08)	−0.12 (−0.29, 0.04)
Ethnicity (ref: Chinese)						
Malay	0.94 (0.64, 1.25)	1.04 (0.66, 1.41)	0.42 (0.20, 0.64)	0.42 (0.14, 0.70)	0.44 (0.29, 0.60)	0.51 (0.33, 0.70)
Indian	0.60 (0.28, 0.92)	0.54 (0.15, 0.94)	0.39 (0.16, 0.62)	0.39 (0.10, 0.69)	0.14 (−0.03, 0.30)	0.09 (−0.11, 0.28)
Child sex (ref: Female)						
Male	−0.05 (−0.27, 0.18)	0.00 (−0.27, 0.27)	−0.06 (−0.22, 0.11)	0.01 (−0.19, 0.21)	−0.05 (−0.16, 0.06)	−0.08 (−0.21, 0.05)
Birth order (ref: Second born)						
First born	0.16 (−0.08, 0.41)	0.17 (−0.12, 0.47)	−0.01 (−0.19, 0.17)	−0.03 (−0.24, 0.19)	0.14 (0.02, 0.27)	0.18 (0.04, 0.32)
Monthly household incomes (ref: ≥6000 SGD)						
< 2000 SGD	0.28 (−0.17, 0.73)	0.15 (−0.43, 0.72)	0.41 (0.08, 0.74)	0.38 (−0.04, 0.81)	−0.17 (−0.40, 0.06)	−0.26 (−0.54, 0.01)
2000-3999 SGD	0.20 (−0.16, 0.57)	−0.04 (−0.48, 0.41)	0.22 (−0.05, 0.49)	0.04 (−0.29, 0.37)	−0.05 (−0.24, 0.13)	−0.11 (−0.33, 0.10)
4000-5999 SGD	0.11 (−0.22, 0.44)	0.01 (−0.38, 0.40)	0.10 (−0.14, 0.35)	0.00 (−0.29, 0.29)	0.00 (−0.16, 0.17)	−0.01 (−0.20, 0.18)
Maternal age (ref: ≥35 years)						
< 25 years	0.43 (−0.02, 0.89)	0.28 (−0.37, 0.93)	0.19 (−0.14, 0.53)	0.05 (−0.43, 0.53)	0.23 (0.00, 0.46)	0.19 (−0.12, 0.51)
25-29 years	0.14 (−0.17, 0.45)	−0.12 (−0.58, 0.34)	0.10 (−0.13, 0.33)	−0.03 (−0.37, 0.31)	0.06 (−0.10, 0.22)	−0.05 (−0.28, 0.17)
30-34 years	−0.04 (−0.33, 0.26)	−0.28 (−0.68, 0.12)	−0.04 (−0.26, 0.17)	−0.2 (−0.49, 0.10)	0.03 (−0.12, 0.18)	−0.06 (−0.25, 0.14)
Maternal education (ref: University)						
Post-secondary	0.41 (0.10, 0.73)	0.42 (0.03, 0.81)	0.30 (0.07, 0.53)	0.22 (−0.07, 0.51)	0.15 (−0.01, 0.31)	0.24 (0.05, 0.43)
Primary or Secondary	0.34 (−0.03, 0.72)	0.59 (0.12, 1.05)	0.21 (−0.07, 0.48)	0.30 (−0.05, 0.64)	0.14 (−0.05, 0.33)	0.29 (0.06, 0.52)
Maternal daily television viewing time (ref: <1 h)						
1-2 h	0.04 (−0.31, 0.39)	−0.04 (−0.46, 0.38)	0.17 (−0.08, 0.43)	0.09 (−0.22, 0.40)	−0.09 (−0.27, 0.08)	−0.14 (−0.34, 0.06)
2-3 h	0.19 (−0.18, 0.56)	0.04 (−0.41, 0.50)	0.24 (−0.02, 0.51)	0.16 (−0.17, 0.50)	0.01 (−0.18, 0.20)	−0.10 (−0.32, 0.12)
≥ 3 h	0.77 (0.42, 1.12)	0.70 (0.28, 1.13)	0.65 (0.39, 0.90)	0.63 (0.32, 0.95)	0.16 (−0.02, 0.34)	0.06 (−0.15, 0.26)
Paternal age (ref: ≥40 years)						
< 30 years	-	0.52 (−0.05, 1.08)	-	0.42 (0.01, 0.84)	-	0.06 (−0.22, 0.33)
30-34 years	-	>0.62 (0.14, 1.11)	-	0.40 (0.04, 0.76)	-	0.27 (0.03, 0.50)
35-39 years	-	0.32 (−0.12, 0.75)	-	0.27 (−0.05, 0.6)	-	0.03 (−0.18, 0.24)
Paternal education (ref: University)						
Primary or Secondary	-	0.10 (−0.35, 0.55)	-	0.11 (−0.23, 0.44)	-	0.02 (−0.19, 0.24)
Post-secondary	-	−0.01 (−0.41, 0.4)	-	−0.01 (−0.31, 0.29)	-	0.05 (−0.15, 0.25)
Paternal daily television viewing time (ref: <1 h)						
1-2 h	-	0.04 (−0.29, 0.36)	-	−0.08 (−0.32, 0.16)	-	0.12 (−0.04, 0.28)
2-3 h	-	0.46 (0.03, 0.89)	-	0.29 (−0.03, 0.61)	-	0.25 (0.04, 0.46)
≥ 3 h	-	0.40 (−0.03, 0.83)	-	0.29 (−0.03, 0.60)	-	0.14 (−0.06, 0.35)

Values are regression coefficients (95% CI) of multivariable linear mixed-effect models. Models were adjusted for all the variables displayed in the table

Interactions were observed between maternal age and child age (time point) and both television (*p* = 0.07) and hand-held device (*p* = 0.05) viewing time as outcomes (Additional file 2: Figure S1). Television viewing time at 2 years was longer in children of mothers aged <30 years than those of mothers ≥30 years, whereas no differences were observed at 3 years. The interaction pattern was opposite for hand-held device viewing time; no differences by maternal age were observed at age 2 years, whereas viewing time at age 3 years was longest in children of mothers <25 years. No other factors were significantly associated with the change in SVT between ages 2 and 3 years.

No interaction was found between child sex and ethnicity, nor between child sex and parental television viewing time. In logistic regression models, the predictors of total SVT ≥2 and ≥4 h/day were similar to those observed in the linear mixed-effect regressions (not shown). Finally, sensitivity analyses using multiple imputation for missing paternal viewing time showed comparable results (not shown).

## Discussion

In our tri-ethnic Singaporean mother-offspring cohort, we found a substantial amount of reported total SVT in early childhood, mainly attributable to television and hand-held devices, whereas computers were used by few children. An increase in total SVT was seen from age 2 to 3 years, largely owing to an increase in hand-held device viewing time. SVT tracked moderately from age 2 to 3 years for all types of screen devices. We identified several predictors of SVT; Malay and Indian ethnicity, maternal education less than university level, and higher parental television viewing time were significantly and independently associated with longer total SVT. Predictors of device-specific viewing time differed somewhat. Low household income and high maternal daily television viewing time were associated with longer television viewing time, whereas being a first-born was associated with longer hand-held device viewing time. Finally, younger maternal age was a significant predictor of an increase in SVT between 2 and 3 years.

To the best of our knowledge, this is the first study to use a longitudinal design to assess SVT and its predictors in Asians children. The GUSTO cohort is based in Singapore, a high-income country where screen use is among the highest globally [16]. At 2 years of age, about 75% of our study children exceeded the recent AAP recommendations for 2 and 3 year old children [15]. Similarly to previous reports in older children, we found that SVT tracks moderately between 2 and 3 years [23]. An increase in SVT between 2 and 3 years was mostly due to an increase in hand-held device SVT, consistent with findings from a cross-sectional study among Singaporean children aged 2 years and below. In that study, Goh et al. also reported that greater SVT at older ages was mainly attributable to use of hand-held devices [19]. Our study also corroborates their finding that young Malay and Indian children engage in longer SVT than Chinese children, even after controlling for other sociodemographic variables. Although the relationship between ethnicity and childhood SVT may be country- and context-specific, the available literature suggests that children from ethnic majorities spend less time on screen devices [18, 24–26].

Consistent with other studies on children of the same age, we did not observe a sex difference in SVT [14, 19, 24, 27–29]. However, it has been reported that boys spend more time on screen devices after age 4-5 years, suggesting that a difference by sex may emerge with age [30–32]. Among the studies that have examined birth order or number of siblings, most were conducted before 2010, when tablets and smartphones were not widely available [24, 28, 33–36]. Other sociodemographic factors associated with longer total or device-specific SVT in our study were low household income, maternal education less than university level, and paternal age less than 35 years. Paternal education was, however, not associated with SVT. Other studies have found similar associations, although the findings remain mixed and likely population-specific [18, 37]. Interestingly, we found that maternal age was associated with change in total and device-specific SVT between 2 and 3 years. Children of mothers younger than 25 years engaged in longer television watching at 2 years than did their counterparts, but from 2 to 3 years, they had a larger increase in hand-held device use. This finding may reflect changing parenting patterns of the so-called Generation Y, who grew up with television and now have switched to more recent digital technologies like tablets and smartphones.

Several parental behavioral predictors were also observed. Maternal television viewing time ≥ 3 h/day was among the strongest predictors of children’s total SVT and television viewing time, but not of hand-held device viewing time. Paternal television viewing time ≥ 2 h/day was associated, although less strongly, with total and device-specific SVT. Previous studies have reported that the presence of frequent screen users in the household is associated with children’s screen behavior [18, 36, 37]. Altogether, the evidence suggests that children’s screen use behavior is strongly influenced by parental behavior. Targeting parental behavior in early childhood may be a potentially effective avenue for interventions aimed at reducing children’s SVT.

Strengths of our study include its longitudinal design, wide range of sociodemographic and behavioral predictors measured a priori and assessment of SVT at two ages, a clear advantage over cross-sectional studies. We also assessed exposure to different types of screen devices, including hand-held devices, which has been uncommon in previous studies. We treated SVT as a continuous variable, thus providing greater statistical power vis-à-vis categorized variables. Study limitations include reliance on SVT reported by parents, who may overlook the child’s screen use when the parents are not at home or during daycare. Whilst the questionnaire we administered has been used in other studies [38], its validity and reliability are unknown. Moreover, it includes no information about the context of screen use (number of screens, parental knowledge on the recommendations, and rule-setting practices), nor on the viewing content. Contextual information is of importance when developing and testing interventions, as highlighted by the recent AAP guidelines that now included media content as an additional target. However, assessing both objective and qualitative SVT measures is challenging and costly in large epidemiological studies. We assessed maternal television viewing time during pregnancy, but it may have changed after birth. Finally, the GUSTO cohort is not representative of the entire Singaporean population. Malay and Indian families were overrepresented purposely at inclusion. GUSTO mothers were also less likely to hold a university degree than the women from the general population of the same age range [39]. The SVT figures observed in our study may therefore differ somewhat from those at the national level.

## Conclusions

In conclusion, we observed a substantial total SVT in Singaporean children at ages 2 and 3 years. Significant predictors of total SVT included ethnicity, maternal education and parental television viewing, suggesting potential targets for health promotion activities. We observed stable television viewing time between 2 and 3 years, whereas hand-held device viewing time increased over time, particularly in children of young mothers. Intervention studies targeting parental screen behavior in early childhood may be warranted: for example, by testing the impact of recommending that parents who watch screens frequently limit their own SVT while their toddler is present. Future interventions should also take into account the evolution of screen usage, which tends to move from television to smartphones and tablets [40].

## Additional files

Additional file 1: Table S1. Summary of the measurement method and time point of assessment of the variables examined as predictors of screen viewing time in 2- and 3-year-old children from the GUSTO cohort study. (DOCX 12 kb)

Additional file 2: Figure S1. Television and hand-held devices viewing time (h/day) at ages 2 and 3 years according to maternal age in children from the GUSTO cohort study. Values are means ± SE, and *p*-values are for the interaction term between maternal age and child age, with viewing time as outcome. (PNG 25 kb)

## Abbreviations

AAP: American Academy of Pediatrics
GUSTO: Growing Up in Singapore Towards healthy Outcomes
SVT: Screen viewing time
