# Supplementary material for: Predictors of screen viewing time in young Singaporean children: the GUSTO cohort
Source: Int J Behav Nutr Phys Act. 2017 Sep 5;14:112. doi: 10.1186/s12966-017-0562-3 (PMC5584344; doi:10.1186/s12966-017-0562-3)
Supplement: Supplementary file 1 — Summary of the measurement method and time point of assessment of the variables examined as predictors of screen viewing time in 2- and 3-year-old children from the GUSTO cohort study. (DOCX 12 kb) [file 12966_2017_562_MOESM1_ESM.docx]

**Additional file 1: Table 1** Summary of the measurement method and time point of assessment of the variables examined as predictors of screen viewing time in 2- and 3-year-old children from the GUSTO cohort study.

| Predictor variables | Method of measurement | Time point of assessment |
| --- | --- | --- |
| Ethnicity | Interviewer-administered questionnaire | Enrolment (<14 weeks’ gestation) |
| Maternal age | Interviewer-administered questionnaire | Enrolment (<14 weeks’ gestation) |
| Paternal age | Interviewer-administered questionnaire | Enrolment (<14 weeks’ gestation) |
| Maternal education | Interviewer-administered questionnaire | Enrolment (<14 weeks’ gestation) |
| Marital status | Interviewer-administered questionnaire | Enrolment (<14 weeks’ gestation) |
| Maternal place of birth | Interviewer-administered questionnaire | Enrolment (<14 weeks’ gestation) |
| Accommodation type | Interviewer-administered questionnaire | Enrolment (<14 weeks’ gestation) |
| Monthly household income | Interviewer-administered questionnaire | Enrolment (<14 weeks’ gestation) |
| Maternal pre-pregnancy BMI (weight/height²) | Interviewer-administered questionnaire (weight)  Measured (height) | Enrolment (<14 weeks’ gestation) for weight  Second trimester pregnancy visit for height |
| Tobacco consumption | Interviewer-administered questionnaire | Second trimester pregnancy visit (26-28 weeks’ gestation) |
| Alcohol consumption | Interviewer-administered questionnaire | Second trimester pregnancy visit (26-28 weeks’ gestation) |
| Maternal TV viewing time | Interviewer-administered questionnaire | Second trimester pregnancy visit (26-28 weeks’ gestation) |
| Maternal physical activity | Interviewer-administered questionnaire | Second trimester pregnancy visit (26-28 weeks’ gestation) |
| Child sex | Extracted from medical records | Delivery |
| Birth order | Extracted from medical records | Delivery |
| Paternal education | Interviewer-administered questionnaire | Postnatal clinic visit (24 or 36 months) |
| Paternal television viewing time | Interviewer-administered questionnaire | Postnatal clinic visit (24 or 36 months) |
| Paternal BMI | Derived from measured weight and height | Postnatal clinic visit (24 or 36 months) |
