# Supplementary figures and images for: Predictors of screen viewing time in young Singaporean children: the GUSTO cohort
Source: Int J Behav Nutr Phys Act. 2017 Sep 5;14:112. doi: 10.1186/s12966-017-0562-3 (PMC5584344; doi:10.1186/s12966-017-0562-3)

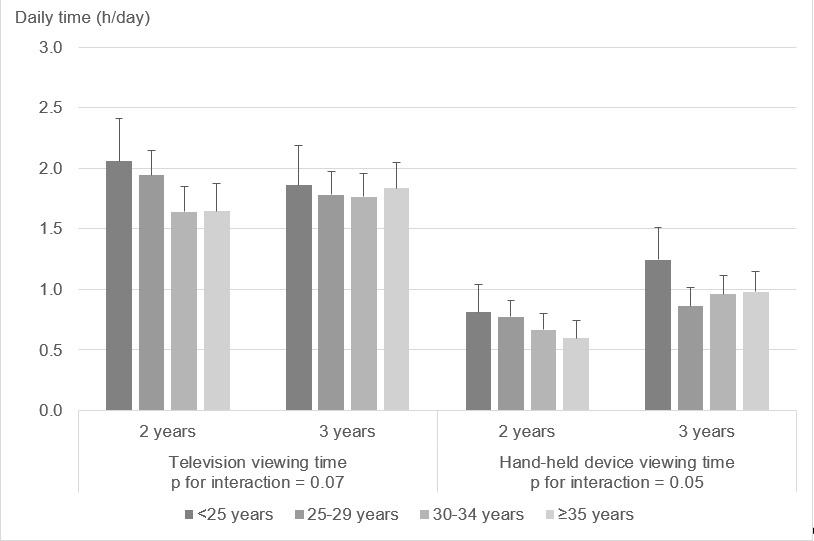

Supplement: Supplementary file 2 — Television and hand-held devices viewing time (h/day) at ages 2 and 3 years according to maternal age in children from the GUSTO cohort study. Values are means ± SE, and p-values are for the interaction term between maternal age and child age, with viewing time as outcome. (PNG 25 kb) [file 12966_2017_562_MOESM2_ESM.png]
